# Clinical characteristics of MOG antibody-positive anti-NMDAR encephalitis: a single-center retrospective study

**DOI:** 10.3389/fneur.2026.1742531

**Published:** 2026-01-27

**Authors:** Xuan Zou, Guan-en Zhou

**Affiliations:** 1 Department of Neurology, Tianjin Huanhu Hospital, Tianjin University, Tianjin, China; 2 Clinical College of Neurology, Neurosurgery and Neurorehabilitation, Tianjin Medical University, Tianjin, China; 3 Department of Neurology, Tianjin Huanhu Hospital, Nankai University, Tianjin, China; 4 Tianjin Key Laboratory of Cerebral Vascular and Neurodegenerative Diseases, Tianjin, China

**Keywords:** anti-NMDAR encephalitis, clinical characteristics, MOG antibody, overlapping demyelinating syndrome, retrospective study

## Abstract

**Objective:**

This study was designed to clarify the clinical characteristics of myelin oligodendrocyte glycoprotein (MOG) antibody-positive anti-N-methyl-D-aspartate receptor (anti-NMDAR) encephalitis.

**Methods:**

This was a single-center retrospective study. Patients with anti-NMDAR encephalitis hospitalized at Tianjin Huanhu Hospital were included in the study. The observation group consisted of patients with anti-NMDAR encephalitis who tested positive for serum MOG-antibodies [MOG-Ab (+)], while the reference group included patients who tested negative for serum MOG-antibodies [MOG-Ab (–)]. Clinical data were collected from both groups and statistical methods were employed to analyze the differences between the two groups.

**Results:**

This study enrolled 48 patients (*n* = 48) with anti-NMDAR encephalitis, comprising eight cases (*n* = 8, 16.67%) in the MOG-Ab (+) group and 40 cases (*n* = 40, 83.33%) in the MOG-Ab (–) group. The proportion of male patients in the MOG-Ab (+) group was significantly higher than that in the MOG-Ab (–) group (87.50 vs. 40.00%, χ*2* = 4.274, *P* = 0.039). Patients in the MOG-Ab (+) group frequently experienced headaches, which occurred more often than in the MOG-Ab (–) group (75.00 vs. 25.00%, χ*2* = 5.419, *P* = 0.020). The median white blood cell (WBC) count in the cerebrospinal fluid (CSF) of the MOG-Ab (+) group was 125.00 (65.00, 155.00) × 10^6^/L, representing a rate that is 12.5 times higher than that of the MOG-Ab (–) group, indicating more pronounced immune inflammatory response in the CSF of the MOG-Ab (+) group (*Z* = −3.320, *P* = 0.000). Additionally, the MOG-Ab (+) group exhibited a higher proportion of leptomeningeal enhancement (37.50 vs. 2.50%, *P* = 0.012) and cortical lesions (87.50 vs. 40.00%, χ*2* = 4.274, *P* = 0.039) on MRI. Recurrence occurred in 50.0% of patients in the MOG-Ab (+) group within 1 year of discharge follow-up, compared to only 12.50% in the MOG-Ab (–) group (χ*2* = 3.938, *P* = 0.047).

**Conclusion:**

For patients with anti-NMDAR encephalitis presenting with headaches, if there is a significant increase in CSF-WBC count, coupled with abnormal MRI signals in the leptomeninges or cortex, it is recommended to test for MOG antibodies. This is particularly crucial for male patients. For those with positive double antibodies, more aggressive long-term immunosuppressive therapy may be necessary to prevent recurrence.

## 1 Introduction

Anti-N-methyl-D-aspartate receptor (anti-NMDAR) encephalitis is the first identified autoimmune encephalitis targeting neuronal cell surface antibodies and is also the most common type. The disease arises when anti-NMDAR antibodies bind to extracellular conformational epitopes in the NR1/NR2 heteromers of the NMDAR. Anti-NMDAR encephalitis is closely associated with ovarian teratomas and is more frequently observed in young women (1). The primary clinical symptoms include epilepsy, mental disorders, cognitive impairment, involuntary movements, and autonomic dysfunction.

It is worth noting that some patients with anti-NMDAR encephalitis may also have other central nervous system (CNS) antibodies, including myelin oligodendrocyte glycoprotein (MOG) antibodies, aquaporin-4 (AQP4) antibodies, and glial fibrillary acidic protein (GFAP) antibodies. This phenomenon is referred to as overlapping anti-NMDAR encephalitis and demyelinating syndrome (2). Among these antibodies, MOG antibodies are the most prevalent, and this condition is described in the literature as the myelin oligodendrocyte glycoprotein antibody and n-methyl-d-aspartate receptor antibody overlapping syndrome (MNOS) (3) or overlapping syndrome of MOG antibody-associated disease (MOGAD) and anti-NMDAR encephalitis (NMDARE) (4). A meta-analysis of 25 studies found that 16.2% of patients with anti-NMDAR encephalitis also presented with a demyelinating syndrome, with 94.5% of these cases classified as MOGAD (2). This indicates that the coexistence of anti-NMDAR encephalitis with MOG antibodies is not uncommon in clinical practice.

MOGAD presents with diverse clinical manifestations, including optic neuritis, cortical encephalitis, brainstem encephalitis, and myelitis. What impact does the presence of MOG antibodies have on anti-NMDAR encephalitis? Currently, literature primarily describes the clinical symptoms of patients with overlap syndrome, but there is a lack of systematic and comprehensive comparisons between individuals who are double antibody positive and those with isolated anti-NMDAR encephalitis (2, 5). This study attempts to explore the clinical differences between them. By clarifying these differences, we can facilitate the prompt identification of overlapping demyelinating syndromes, which may have significant implications for clinical diagnosis and treatment.

## 2 Materials and methods

### 2.1 Sample source

This was a single-center retrospective study. We searched and collected patients with anti-NMDAR encephalitis who were hospitalized at Tianjin Huanhu Hospital, affiliated with Tianjin University, from January 2020 to October 2024. All patients underwent testing for anti-NMDAR antibodies in cerebrospinal fluid (CSF) and MOG antibodies in serum using indirect immunofluorescence assay (IIF), including cell-based assay (CEA) and tissue-based assay (TBA).

### 2.2 Inclusion criteria and exclusion criteria

#### 2.2.1 Inclusion criteria

All patients were aged ≥18 years, had positive CSF anti-NMDAR antibodies and met the diagnostic criteria for anti-NMDAR encephalitis (6), and the patients or their families agreed to be enrolled. The observation group consisted of patients with anti-NMDAR encephalitis who had positive serum MOG antibodies [MOG-Ab (+)], while the reference group consisted of patients with negative serum MOG antibodies [MOG-Ab (–)].

#### 2.2.2 Exclusion criteria

Patients with incomplete clinical data were excluded from the study.

### 2.3 Clinical data collection

#### 2.3.1 General information

Included patient age and gender.

#### 2.3.2 Predisposing factors

Whether there was a pre-infection before the onset of the disease and whether a concurrent tumor was present. Pre-infection referred to infection symptoms experienced within 4 weeks prior to the onset of the patient's illness, including fever, rhinorrhea, sore throat, and similar manifestations. This category also encompassed a documented history of viral encephalitis within 3 months or the detection of viral infection through Next-Generation Sequencing (NGS) of CSF. All patients underwent tumor screening, including tumor biomarker tests as well as computed tomography (CT) or ultrasound examinations targeting visceral organs.

#### 2.3.3 Clinical manifestations

Included the top 4 main clinical symptoms with the highest incidence rate, as well as the top 5 complications. The clinical symptoms included headache, epilepsy, psychiatric symptoms, and cognitive impairment. The first three symptoms were primarily evaluated based on clinical manifestations, while cognitive impairment was assessed using the Mini-Mental State Examination (MMSE), adjusted for the patient's educational level. The complications referred to secondary diseases that might arise during the course of encephalitis, including infection, electrolyte disturbance, hypoproteinemia, liver or renal function injury, and stress ulcer.

#### 2.3.4 Magnetic resonance imaging (MRI)

Included the presence of MRI lesions, cortical lesions, and leptomeningeal enhancement. We primarily evaluated the fluid-attenuated inversion recovery (FLAIR)-weighted sequence, contrast-enhanced T1-weighted sequence, and contrast-enhanced FLAIR-weighted sequence using a 3.0T MRI.

#### 2.3.5 CSF parameters

Included CSF white blood cell count, CSF protein levels, and CSF-IgG concentration.

#### 2.3.6 Severity of illness

Glasgow Coma Scale (GCS) score upon admission.

#### 2.3.7 Treatment status

Whether the patient received multiple first-line immunotherapy treatments, including intravenous immunoglobulin (IVIG), intravenous methylprednisolone (IVMP) and plasma exchange (PE); whether the patient required second-line immunotherapy, such as rituximab or ofatumumab.

#### 2.3.8 Outcome

The evaluation of patient improvement during the acute phase; the recurrence status of patients within 1 year, noting that all patients continued to take gradually reduced oral corticosteroids after discharge. Recurrence was defined as the new onset or worsening of symptoms by ≥1 point on the modified Rankin scale (mRS) following a period of at least 2 months of improvement or stabilization (7, 8).

### 2.4 Statistical analysis methods

In this study, continuous variables did not follow a normal distribution. Therefore, median and quartile range [Median (Quartile Range)] were used to describe their central and discrete trends, and non-parametric tests were used to analyze the differences between the two groups. For categorical variables, this study used the number of cases (composition ratio) [n (%)] to describe and analyzed the differences between the two groups using chi-square test or Fisher's exact test. *P* < 0.05 was considered statistically significant.

## 3 Results

This study enrolled 48 patients (*n* = 48) with anti-NMDAR encephalitis who met the inclusion criteria, comprising eight cases (*n* = 8, 16.67%) in the MOG-Ab (+) group and 40 cases (*n* = 40, 83.33%) in the MOG-Ab (–) group. No patient met the exclusion criteria. The median serum MOG antibody titer in the MOG-Ab (+) group was 1:32 (1:10, 1:100). The comparison between the two groups is presented in Table 1.

**Table 1:** Comparison between two groups.

**Variable**	**MOG-Ab (+) group (*n* = 8)**	**MOG-Ab (–) group (*n* = 40)**	**Statistics**	***P***
**General information**				
Age [year, median (quartile range)]	33.50 (24.50, 38.25)	36.50 (24.50, 54.50)	−0.997	0.333^a^
Male patient [n (%)]	7 (87.50)	16 (40.00)	4.274	0.039^b^
**Predisposing factors**				
Pre-infection [n (%)]	2 (25.00)	21 (52.50)	1.069	0.301^b^
Concurrent tumor [n (%)]	0 (0.00)	6 (15.00)	0.343	0.558^b^
**Clinical manifestations [n (%)]**				
Clinical symptoms				
Headache	6 (75.00)	10 (25.00)	5.419	0.020^b^
Epilepsy	5 (62.50)	17 (42.50)	0.420	0.517^b^
Psychiatric symptoms	4 (50.00)	27 (67.50)	0.291	0.589^b^
Cognitive impairment	0 (0.00)	15 (37.50)	2.793	0.095^b^
Complications [n (%)]	7 (87.50)	27 (67.50)	0.504	0.478^b^
Infection	4 (50.00)	17 (42.50)	0.000	1.000^b^
Electrolyte disturbance	4 (50.00)	14 (35.00)	0.160	0.689^b^
Hypoproteinemia	4 (50.00)	12 (30.00)	0.469	0.494^b^
Liver or renal function injury	3 (37.50)	14 (35.00)	0.000	1.000^b^
Stress ulcer	2 (25.00)	9 (22.50)	0.000	1.000^b^
**Brain MRI [n (%)]**				
Presence of MRI lesion	7 (87.50)	23 (57.50)	1.440	0.230^b^
Cortical lesion	7 (87.50)	16 (40.00)	4.274	0.039^b^
Leptomeningeal enhancement	3 (37.50)	1 (2.50)	-	0.012^c^
**CSF parameters**				
CSF-WBC count (×10^6^/L)	125.00 (65.00, 155.00)	10.00 (6.00, 54.50)	−3.320	0.000^a^
CSF protein level (g/L)	0.6350 (0.4900, 0.8925)	0.4800 (0.3375, 0.7000)	−1.536	0.126^a^
CSF-IgG level (mg/L)	53.5500 (40.4500, 66.2000)	54.4500 (30.6500, 86.9250)	−0.194	0.860^a^
**Severity of illness**				
GCS score [median (quartile range)]	14.50 (9.25, 15.00)	14.00 (10.25, 15.00)	−0.244	0.818^a^
**Treatment [n (%)]**				
Multiple first-line immunotherapies	6 (75.00)	26 (65.00)	0.019	0.891^b^
Second-line immunotherapy	3 (37.50)	7 (17.50)	0.632	0.427^b^
**Outcome [n (%)]**				
Improvement rate in acute phase	6 (75.00)	32 (80.00)	0.000	1.000^b^
Recurrence within 1 year of follow-up	4 (50.00)	5 (12.50)	3.938	0.047^b^

Statistics: a: nonparametric test; b: chi-square test; c: Fisher's exact test

MOG, Myelin oligodendrocyte glycoprotein; CSF, cerebrospinal fluid; IgG, glycoprotein G; MRI, magnetic resonance imaging; WBC, white blood cell; GCS, Glasgow coma scale.

### 3.1 General Information

The median age of the MOG-Ab (+) group was 33.50 (24.50, 38.25) years, while the MOG-Ab (–) group was 36.50 (24.50, 54.50) years, with no significant difference between the two groups (*Z* = −0.997, *P* = 0.333).

In the MOG-Ab (+) group, there were seven male patients (87.50%) and only one female patient (12.50%). In contrast, the MOG-Ab (–) group consisted of 16 male patients (40.00%) and 24 female patients (60.00%). The proportion of male patients in the MOG-Ab (+) group was significantly higher than that in the MOG-Ab (–) group (χ*2* = 4.274, *P* = 0.039).

### 3.2 Predisposing factors

In the MOG-Ab (+) group, two patients (25.00%) had a history of pre-infection, compared to 21 patients (52.50%) in the MOG-Ab (–) group. Although the proportion of pre-infection was higher in the MOG-Ab (–) group, the difference was not statistically significant (χ*2* = 1.069, *P* = 0.301).

The MOG-Ab (+) group had no patient with concurrent tumor (0.00%), while the MOG-Ab (–) group included six patients (15.00%) with tumors (four cases of ovarian teratomas, one case of uterine cancer, and one case of lung cancer). There was no significant difference in tumor occurrence between the two groups (χ*2*0 = .343*, P*=0.558).

### 3.3 Clinical manifestations

The clinical symptoms in the MOG-Ab (+) group included 6 cases (75.00%) with headaches, five cases (62.50%) with epilepsy, and four cases (50.00%) with psychiatric symptoms, with no significant cognitive impairment patients observed (0.00%). In the MOG-Ab (–) group, the clinical symptoms included 27 patients with psychiatric symptoms (67.50%), 17 patients with epilepsy (42.50%), 15 patients with cognitive impairment (37.50%), and 10 patients with headaches (25.00%). The incidence of headaches in the MOG-Ab (+) group was significantly higher than in the MOG-Ab (–) group (χ*2* = 5.419, *P* = 0.020). However, there were no significant differences in the rates of epilepsy (χ*2* = 0.420, *P* = 0.517), psychiatric symptoms (χ*2* = 0.291, *P* = 0.589), or cognitive impairment (χ*2* = 2.793, *P* = 0.095).

In the MOG-Ab (+) group, seven patients (87.50%) experienced complications. Among them, there were four cases (50.00%) of infections, which included three cases of pneumonia, and one case of urinary tract infection. Additionally, the group reported four cases (50.00%) of electrolyte imbalance, four cases (50.00%) of hypoalbuminemia, three cases (37.50%) of liver or kidney dysfunction, and two cases (25.00%) of stress ulcers. In the MOG-Ab (–) group, 27 patients (67.50%) had complications. Among them, there were 17 cases (42.50%) of infections, which included 16 cases of pneumonia, and one case of urinary tract infection. The group also experienced 14 cases (35.00%) of electrolyte disorders, 14 cases (35.00%) of liver or kidney dysfunction, 12 cases (30.00%) of hypoalbuminemia, and nine cases (22.50%) of stress ulcers. There were no significant differences in the incidence of overall complications (χ*2* = 0.504, *P* = 0.478), infections (χ*2* = 0.000, *P* = 1.000), electrolyte imbalances (χ*2* = 0.160, *P* = 0.689), hypoalbuminemia (χ*2* = 0.469, *P* = 0.494), liver or kidney dysfunction (χ*2* = 0.000, *P* = 1.000), or stress ulcers (χ*2* = 0.000, *P* = 1.000) between the two groups.

### 3.4 Brain MRI

**Figure 1:**
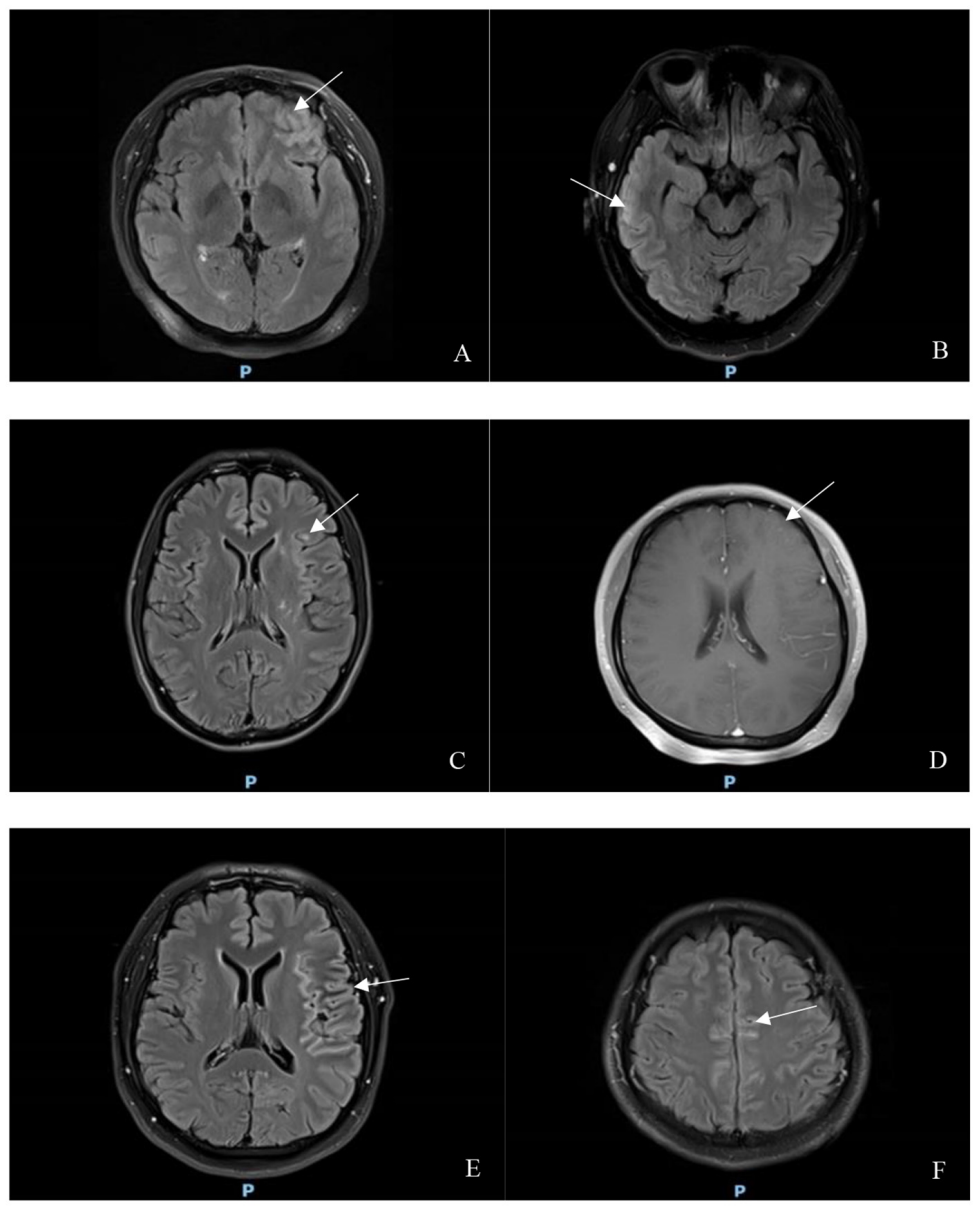
**(A)** shows a high FLAIR signal in the left forehead; **(B)** shows a high FLAIR signal in the right temporal lobe; **(C)** shows a high FLAIR signal in the left temporal and insular lobes; **(D)** shows left frontal leptomeningeal enhancement on the contrast-enhanced T1-weighted sequence; **(E)** shows FLAIR enhancement changes in the left temporal leptomeninges; **(F)** shows FLAIR enhancement changes in the leptomeninges adjacent to the midline of both frontal and parietal regions.

In the MOG-Ab (+) group, seven patients (87.50%) exhibited lesions on brain MRI, all of which were cortical lesions (typical images are shown in Figures 1A–C). Additionally, three patients (37.50%) demonstrated leptomeningeal enhancement (see Figures 1D–F). In the MOG-Ab (–) group, 23 patients (57.50%) had lesions on brain MRI, including16 cases (40.00%) with cortical lesions, and only one case (2.50%) showing leptomeningeal enhancement. Although there was no significant difference in the presence of brain MRI lesions between the two groups (χ*2* = 1.440, *P* = 0.230), the MOG-Ab (+) group showed a higher likelihood of leptomeningeal enhancement (*P* = 0.012) and a greater incidence of cortical lesions (χ*2* = 4.274, *P* = 0.039).

### 3.5 CSF parameters

The median WBC count in CSF of the MOG-Ab (+) group was 125.00 (65.00, 155.00) × 10^6^/L, while that of the MOG-Ab (–) group was 10.00 (6.00, 54.50) × 10^6^/L. There was a significant difference between the two groups (*Z* = −3.320, *P* = 0.000).

The median of CSF protein level in the MOG-Ab (+) group was 0.6350 (0.4900, 0.8925) g/L, compared to 0.4800 (0.3375, 0.7000) g/L in the MOG-Ab (–) group. The median CSF-IgG level in the MOG-Ab (+) group was 53.5500 (40.4500, 66.2000) mg/L, while in the MOG-Ab (–) group it was 54.4500 (30.6500, 86.9250) mg/L. There was no significant difference in CSF protein (*Z* = −1.536, *P* = 0.126) or CSF-IgG (*Z* = −0.194, *P* = 0.860) levels between the two groups.

### 3.6 Severity of illness

The median GCS score upon admission for the MOG-Ab (+) group was 14.50 (9.25, 15.00) points, while for the MOG-Ab (–) group it was 14.00 (10.25, 15.00) points. Although the degree of consciousness impairment in the MOG-Ab (+) group was milder than that in the MOG-Ab (–) group, the difference was not statistically significant (*Z* = −0.244, *P* = 0.818).

### 3.7 Treatment

In the MOG-Ab (+) group, six patients (75.00%) required multiple first-line immunotherapies, while in the MOG-Ab (–) group, 26 patients (65.00%) required multiple first-line immunotherapies. Additionally, three patients (37.50%) in the MOG-Ab (+) group needed second-line immunotherapy, compared to seven patients (17.50%) in the MOG-Ab (–) group. Although patients in the MOG-Ab (+) group appeared to require second-line treatment more frequently than those in the MOG-Ab (–) group, there was no difference between the two groups regarding the need for first-line (χ*2* = 0.019, *P* = 0.891) or second-line (χ*2* = 0.632, *P* = 0.427) treatment.

### 3.8 Outcome

In the MOG-Ab (+) group, six patients (75.00%) showed improvement after treatment, while two patients (25.00%) did not show any improvement. In the MOG-Ab (–) group, 32 cases (80.00%) improved, whereas eight cases (20.00%) showed no improvement after treatment. There was no significant difference in the acute phase outcomes between the two groups (χ*2* = 0.000, *P* = 1.000).

During the 1-year follow-up, four patients (50.00%) in the MOG-Ab (+) group experienced recurrence, compared to five patients (12.50%) in the MOG-Ab (–) group. The recurrence rate of the MOG-Ab (+) group was significantly higher than that in the MOG-Ab (–) group (χ*2* = 3.938, *P* = 0.047).

### 3.9 Conclusion

A total of 48 patients with anti-NMDAR encephalitis were included in this study, of which 8 (16.67%) tested positive for serum MOG antibody. This incidence rate is consistent with that reported in the literature (2). The MOG-Ab (+) group was predominantly male (χ*2* = 4.274, *P* = 0.039) and exhibited a higher prevalence of headaches (χ*2* = 5.419, *P* = 0.020). Additionally, this group showed greater susceptibility to leptomeningeal (*P* = 0.012) and cortical involvement (χ*2* = 4.274, *P* = 0.039), significantly elevated WBC counts in CSF (*Z* = −3.320, *P* = 0.000), and a higher likelihood of recurrence during follow-up (χ*2* = 3.938, *P* = 0.047). No significant differences were observed between the two groups in terms of age, predisposing factors, complications, disease severity, treatment, and acute phase outcomes.

## 4 Discussion

The mechanism underlying the overlap between anti-NMDAR encephalitis and MOG antibodies remained unclear. NMDAR and MOG could coexist on oligodendrocytes, and simultaneous or sequential stimulation of these two antigens might contribute to the presence of both antibodies (9). Another explanation was that the disruption of the blood-brain barrier (BBB) might have exposed multiple antigens, resulting in the formation of overlapping antibodies. Notably, a significant portion of patients with anti-NMDAR encephalitis initially did not present with MOG antibodies; however, as the disease progressed, MOG antibodies might emerge, accounting for approximately 50% of cases (5). Thus, it was plausible that anti-NMDAR encephalitis exacerbated BBB disruption, leading to increased exposure to MOG antigens.

The majority of MOG-Ab (+) patients with anti-NMDAR encephalitis included in this study were young males, accounting for 87.5% of the cohort, which aligned with findings reported in previous literature (3, 10, 11). One potential explanation for this predominance was that males were more prone to BBB dysfunction (12), leading to increased exposure to multiple antigens. Additionally, it was possible that male patients were more likely to develop MOG antibodies. According to literature, male patients with pure MOGAD accounted for approximately 60% of the total patient population (13–15), whereas female patients were more commonly seen in cases of pure anti-NMDAR encephalitis. Female patients with anti-NMDAR encephalitis were more likely to be associated with tumors, particularly ovarian teratomas, whereas the MOG-Ab (+) group was predominantly composed of male patients. Consequently, the incidence of tumors in the MOG-Ab (+) group was significantly lower than that in the MOG-Ab (–) group (0.00 vs. 15.00%).

The clinical symptoms of patients with dual antibodies were more similar to those of anti-NMDAR (9, 11). Most literature indicated that the most common clinical manifestations in these patients were epilepsy and psychiatric symptoms (2, 3). Reported rates showed that 51%−72.7% of patients with bispecific antibodies experienced seizures, while 45.5%−71.4% exhibited psychiatric symptoms (2). In this study, 62.5% of MOG antibody-positive patients with anti-NMDAR encephalitis experienced epileptic seizures, and 50% had psychiatric symptoms.

However, it was important to note that the most common prevalent symptom in patients with dual antibodies was not epilepsy or psychiatric symptoms, but rather headaches. In this study, 75.00% of patients in the MOG-Ab (+) group experienced headaches, significantly higher than the 25% of patients with isolated anti-NMDAR encephalitis. This clinical symptom was often overlooked by clinical doctors, and there were few reports highlighting its significance. A study from Xuanwu Hospital at Capital Medical University found a significant difference in headaches prevalence between the NMDAR (+)/MOG (+) group and the NMDAR (+)/MOG (–) group (10). Additionally, another study analyzed 12 patients with coexistence of anti-MOG associated encephalitis with seizures (FLAMES) and anti-N-methyl-D-aspartate receptor encephalitis (anti-NMDARe), and 91.67% of these patients experienced headaches (16).

MOG antibody-positive anti-NMDAR encephalitis patients were particularly to headaches, possibly due to the presence of MOG antibodies. Research indicated that approximately 80% of patients with MOG antibody associated-cortical encephalitis would experience headaches (13, 14). In contrast, headaches were not typically considered a primary clinical manifestation of anti-NMDAR encephalitis, which might be due to the rapid masking of headache symptoms by other encephalitis -related symptoms (17).

According to the literature (13, 14), approximately 33.33%−45.45% of patients with MOGAD might present with leptomeningeal enhancement. In this study, 37.50% of MOG antibody-positive anti-NMDAR encephalitis patients exhibited leptomeningeal enhancement, which might contribute to the headaches associated with MOG antibodies. In addition to the frequent involvement of the leptomeninges, this study also found that patients with MOG antibody-positive anti-NMDAR encephalitis had a higher prevalence of cortical involvement, with 87.50% of these patients displaying cortical lesions compared to only 40% in cases of isolated anti-NMDAR encephalitis. A meta-analysis that included 58 patients with dual antibody encephalitis reported that 70.7% of common MRI changes were observed in the cortex or subcortical region (11). Furthermore, another study focusing on patients with dual antibody encephalitis who experienced epileptic seizures found that 100% exhibited high FLAIR signals in the cortex (16). Compared to patients who were negative for anti-MOG antibodies, those with dual antibody encephalitis tended to exhibit more frequent cortical or subcortical lesions (11).

MOG antibody-positive anti-NMDAR encephalitis patients exhibited involvement of the leptomeninges and cortex, leading to more pronounced CSF inflammatory reactions and leptomeningeal irritation symptoms, such as headaches. In this study, the median CSF white blood cell count in patients with dual antibody encephalitis was 125.00 (65.00, 155.00) × 10^6^/L. Previous studies have reported a median CSF white blood cell count of 128 × 10^6^/L in patients with positive dual antibodies (16), while the median count in patients with isolated anti-NMDAR encephalitis in this study was only 10.00 (6.00, 54.50) × 10^6^/L. There was a significant difference in CSF white blood cell count between the dual antibody group and the single anti-NMDAR encephalitis group, with the former demonstrating more severe intracranial immune inflammation (10). The increase in CSF white blood cell count was also closely associated with the presence of MOG antibodies. Given the frequent involvement of the leptomeninges and cortex, the WBC count in the CSF of patients with MOGAD-associated encephalitis could exceed 100 × 10^6^/L (18), while those with isolated anti-NMDAR encephalitis typically exhibited only mild elevations. This highlighted that leptomeningeal and cortical involvement, as well as CSF inflammation, might both be clinical manifestations associated with MOG antibodies.

There was no significant difference in acute treatment and prognosis between patients with MOG antibody-positive anti-NMDAR encephalitis and those with isolated anti-NMDAR encephalitis; however, the recurrence rate in the former group was significantly higher. In this study, the recurrence rate among dual antibody patients was 50% after a 1-year follow-up post-discharge, compared to 12.5% in patients with isolated anti-NMDAR encephalitis. The elevated recurrence rate in dual antibody encephalitis had been widely documented in previous literature (3, 11). A meta-analysis indicated that 63.4% of patients with dual antibody encephalitis experience recurrence (11), and this rate might increase with prolonged follow-up. For instance, at a median follow-up of 23.5 months, 70% of patients experienced recurrence (19). There was even report suggesting that, with an average follow-up time of 25.4 months, the recurrence rate among patients with dual antibody encephalitis could reach 100%, with an average recurrence interval of 6.7 months (5). Although this high recurrence rate did not appear to adversely affect the long-term prognosis of patients (9), it undoubtedly imposed significant economic and psychological burdens.

The increased susceptibility to recurrence in patients with dual antibody encephalitis might be attributed to the presence of significant and difficult-to-manage risk factors that facilitate the production of autoimmune antibodies, such as the breakdown of the BBB and exposure to various antigens. Therefore, they were prone to multiple antibody accumulation during the acute phase and to recurrence during the recovery phase.

## 5 Conclusion

In summary, patients with dual antibody encephalitis often exhibit clinical manifestations typical of anti-NMDAR encephalitis; however, the presence of MOG antibodies can also lead to distinctive features. When patients with anti-NMDAR encephalitis present with notable symptoms of leptomeningeal irritation, such as headaches, along with a significantly elevated CSF white blood cell count and brain MRI findings primarily showing leptomeningeal enhancement and cortical lesions, serum MOG antibody testing should be conducted—particularly in male patients. Early recognition of MOG antibody accumulation and prompt treatment can be beneficial for these patients. Furthermore, given the high recurrence rate in anti-NMDAR encephalitis patients with MOG antibodies, long-term immunosuppressive therapy is recommended (3).

## 6 Deficiency and inspiration

This study is a retrospective analysis, and the results may have been influenced by bias or confounding factors. Additionally, MOG antibody-positive anti-NMDAR encephalitis is a rare disease, resulting in a relatively small sample size for the experimental group, which may influence the statistical outcomes. Further studies should aim to expand the sample size to enhance the accuracy of the results.

## Data Availability

The original contributions presented in the study are included in the article/Supplementary material, further inquiries can be directed to the corresponding author.
