# Supplementary material for: Clinical characteristics of MOG antibody-positive anti-NMDAR encephalitis: a single-center retrospective study
Source: Front Neurol. 2026 Jan 27;17:1742531. doi: 10.3389/fneur.2026.1742531 (PMC12888224; doi:10.3389/fneur.2026.1742531)
Supplement: Supplementary file 1 [file Table_1.DOCX]

Table 1. Comparison between two groups

| Variable | MOG-Ab (+) group  (n=8) | MOG-Ab (-) group  (n=40) | Statistics | *P* |
| --- | --- | --- | --- | --- |
| General information |  |  |  |  |
| Age [year, median (quartile range)] | 33.50 (24.50, 38.25) | 36.50 (24.50, 54.50) | -0.997 | 0.333 ^a^ |
| male patient [n (%)] | 7 (87.50) | 16 (40.00) | 4.274 | 0.039 ^b^ |
| Predisposing factors |  |  |  |  |
| pre-infection [n (%)] | 2 (25.00) | 21 (52.50) | 1.069 | 0.301 ^b^ |
| concurrent tumor [n (%)] | 0 (0.00) | 6 (15.00) | 0.343 | 0.558 ^b^ |
| Clinical manifestations [n (%)] |  |  |  |  |
| clinical symptoms |  |  |  |  |
| headache | 6 (75.00) | 10 (25.00) | 5.419 | 0.020 ^b^ |
| epilepsy | 5 (62.50) | 17 (42.50) | 0.420 | 0.517 ^b^ |
| psychiatric symptoms | 4 (50.00) | 27 (67.50) | 0.291 | 0.589 ^b^ |
| cognitive impairment | 0 (0.00) | 15 (37.50) | 2.793 | 0.095 ^b^ |
| complications [n (%)] | 7 (87.50) | 27 (67.50) | 0.504 | 0.478 ^b^ |
| infection | 4 (50.00) | 17 (42.50) | 0.000 | 1.000 ^b^ |
| electrolyte disturbance | 4 (50.00) | 14 (35.00) | 0.160 | 0.689 ^b^ |
| hypoproteinemia | 4 (50.00) | 12 (30.00) | 0.469 | 0.494 ^b^ |
| liver or renal function injury | 3 (37.50) | 14 (35.00) | 0.000 | 1.000 ^b^ |
| stress ulcer | 2 (25.00) | 9 (22.50) | 0.000 | 1.000 ^b^ |
| Brain MRI [n (%)] |  |  |  |  |
| presence of MRI lesion | 7 (87.50) | 23 (57.50) | 1.440 | 0.230 ^b^ |
| cortical lesion | 7 (87.50) | 16 (40.00) | 4.274 | 0.039 ^b^ |
| leptomeningeal enhancement | 3 (37.50) | 1 (2.50) | - | 0.012 ^c^ |
| CSF parameters |  |  |  |  |
| CSF-WBC count (×10^6^/L) | 125.00 (65.00, 155.00) | 10.00 (6.00, 54.50) | -3.320 | 0.000 ^a^ |
| CSF protein level (g/L) | 0.6350 (0.4900, 0.8925) | 0.4800 (0.3375, 0.7000) | -1.536 | 0.126 ^a^ |
| CSF-IgG level (mg/L) | 53.5500 (40.4500, 66.2000) | 54.4500 (30.6500, 86.9250) | -0.194 | 0.860 ^a^ |
| Severity of illness |  |  |  |  |
| GCS score [median (quartile range)] | 14.50 (9.25，15.00) | 14.00 (10.25，15.00) | -0.244 | 0.818^a^ |
| Treatment [n (%)] |  |  |  |  |
| multiple first-line immunotherapies | 6 (75.00) | 26 (65.00) | 0.019 | 0.891 ^b^ |
| second-line immunotherapy  Outcome [n (%)] | 3 (37.50) | 7 (17.50) | 0.632 | 0.427 ^b^ |
| Improvement rate in acute phase | 6 (75.00) | 32 (80.00) | 0.000 | 1.000 ^b^ |
| recurrence within one year of follow-up | 4 (50.00) | 5 (12.50) | 3.938 | 0.047 ^b^ |

Statistics: a: nonparametric test；b: chi-square test; c: Fisher's exact test. MOG, Myelin oligodendrocyte glycoprotein; CSF, cerebrospinal fluid; IgG, glycoprotein G; MRI, Magnetic Resonance Imaging; WBC, white blood cell; GCS, Glasgow Coma Scale.
